# Prevalence of adult smokers in Brazilian capitals according to socioeconomic deprivation

**DOI:** 10.1590/1980-549720230044

**Published:** 2023-10-20

**Authors:** Regina Tomie Ivata Bernal, Deborah Carvalho Malta, Renato Azeredo Teixeira, Alastair Hay Leyland, Vittal Srinivasa Katikireddi, Elizabeth Bailey Brickley, Elzo Pereira Pinto, Maria Yuri Travassos Ichiara, Mirjam Allik, Ruth Dundas, Mauricio Lima Barreto

**Affiliations:** I Universidade Federal de Minas Gerais, School of Nursing, Department of Maternal-Child Nursing and Public Health – Belo Horizonte (MG), Brazil.; II University of Glasgow, Medical Research Council, Scottish Government Chief Scientist Office, Social and Public Health Sciences Unit, School of Health and Wellbeing – Glasgow, UK.; III London School of Hygiene and Tropical Medicine, Department of Infectious Disease Epidemiology – London, UK.; IV Fundação Oswaldo Cruz, Center for Integration of Data and Knowledge for Health – Salvador (BA) Brazil.

**Keywords:** Health inequities, Social inequity, Prevalence studies, Small-area analysis, Tobacco, Surveys, Iniquidades em saúde, Iniquidade social, Estudos de prevalência, Análise de pequenas áreas, Tabaco, Inquéritos

## Abstract

**Objective:**

To estimate the prevalence of adult smokers in the 26 capitals and the Federal District according to the Brazilian Deprivation Index (*Índice Brasileiro de Privação* – IBP).

**Methods:**

Dataset on smoking were obtained from the Surveillance of Risk and Protective Factors for Noncommunicable Diseases by Survey (Vigitel) system for the 26 capitals and the Federal District, in the period from 2010 to 2013. The IBP classifies the census sectors according to indicators such as: income less than ½ minimum wage, illiterate population and without sanitary sewage. In the North and Northeast regions, the census sectors were grouped into four categories (low, medium, high and very high deprivation) and in the South, Southeast and Midwest regions into three (low, medium and high deprivation). Prevalence estimates of adult smokers were obtained using the indirect estimation method in small areas. To calculate the prevalence ratios, Poisson models are used.

**Results:**

The positive association between prevalence and deprivation of census sector categories was found in 16 (59.3%) of the 27 cities. In nine (33.3%) cities, the sectors with the greatest deprivation had a higher prevalence of smokers when compared to those with the least deprivation, and in two (7.4%) there were no differences. In Aracaju, Belém, Fortaleza, João Pessoa, Macapá and Salvador, the prevalence of adult smokers was three times higher in the group of sectors with greater deprivation compared to those with less deprivation.

**Conclusion:**

Sectors with greater social deprivation had a higher prevalence of smoking, compared with less deprivation, pointing to social inequalities.

## RESUMO

**Objetivo:**

Estimar as prevalências de adultos fumante nas 26 capitais e no Distrito Federal segundo o Índice Brasileiro de Privação.

**Métodos:**

Os dados sobre tabagismo foram obtidos junto ao sistema de Vigilância de Fatores de Risco e Proteção para Doenças Crônicas por Inquérito (Vigitel) para as 26 capitais e o Distrito Federal, no período de 2010 a 2013. O Índice Brasileiro de Privação classifica os setores censitários segundo indicadores como: renda menor que meio salário mínimo, população não alfabetizada e sem esgotamento sanitário. Nas regiões Norte e Nordeste, os setores censitários foram agrupados em quatro categorias (baixa, média, alta e muito alta privação) e, nas regiões Sul, Sudeste e Centro-Oeste, em três (baixa, média e alta privação). As estimativas de prevalências de adultos fumantes foram obtidas pelo método indireto de estimação em pequenas áreas. Para o cálculo das razões de prevalências, empregram-se modelos de Poisson.

**Resultados:**

A associação positiva entre a prevalência e a privação das categorias de setores censitários foi encontrada em 16 (59,3%) das 27 cidades. Em nove (33,3%) cidades, os setores de maior privação apresentaram maior prevalência de fumantes quando comparados aos de menor privação e, em duas (7,4%), não apresentaram diferenças. Em Aracaju, Belém, Fortaleza, João Pessoa, Macapá e Salvador, as prevalências de adultos fumantes foram três vezes maiores no grupo de setores com maior privação em relação aos de menor privação.

**Conclusão:**

Setores de maior privação social apresentaram maiores prevalências de tabagismo, comparados com menor privação, apontando desigualdades sociais.

## INTRODUCTION

According to the World Health Organization (WHO), tobacco is the main risk factor for preventable causes of death and the second largest attributable factor of mortality in the world^1^. Tobacco use is associated with variables such as low income, low education^2^, and living in places with high vulnerabilities^3^.

The place of residence is presented, among the social determinants, as a component strongly shaped by the social position in which it is allocated, showing that the aspects of the physical surroundings of the neighborhood can be important factors for the perpetuation of inequities in health^4,5^. To this end, in addition to considering social aspects, epidemiological research makes use of spatial analysis to identify the influence of spaces related to exposure differentials and inequalities, expanding the understanding of the occurrence of health-related events in populations and in the processes of morbidity and mortality^6–8^.

Acting through research in these intra-urban relationships allows identifying where and how interventions should be carried out, and one of the tools used to understand the relationships between social determinants and health outcomes is geoprocessing, an important strategy in identifying areas of vulnerability^9^.

It is noteworthy that most states lack health information on their population in small areas for formulating local public policy programs, given the high cost of surveys of this nature.

In this sense, the area of statistics has contributed with methods for obtaining reliable estimates for smaller areas, such as regional health, districts or sub-regions, not initially contemplated in the research sampling plans^10^. The indirect estimation method for small areas based on models has been widely used in several areas^9^. This method uses survey data and auxiliary information extracted from the last census, at the lowest level, as predictor variables of the model for estimating the variable of interest in smaller areas^10^.

In 2019, the Center for Integration of Data and Knowledge for Health (*Centro de Integração de Dados e Conhecimentos para Saúde* – CIDACS) in partnership with the University of Glasgow built the deprivation index for Brazil, called the Brazilian Deprivation Index (*Índice Brasileiro de Privação* – IBP), using data from the 2010 demographic census. This index allows to highlight the inequalities of different social groups and the comparison between municipalities and Brazilian regions. The index was built to measure inequalities in the country using a single cutoff point for all of Brazil. This index is presented by quartile, quintile, and vigintile of deprivation^11^.

The use of composite indicators^12–21^, such as the IBP, may support the production of estimates related to risk factors for noncommunicable chronic diseases (NCDs) in smaller areas and, thus, support policies to promote equity^1^. The present study aimed to produce estimates of prevalence of adult smokers, according to the IBP, in the 26 capitals and in the Federal District.

## METHODS

This is an ecological study using data from the Surveillance of Risk and Protective Factors for Chronic Diseases by Telephone Survey (*Vigilância de Fatores de Risco e Proteção para Doenças Crônicas por Inquérito* – Vigitel) system, in the 26 capitals and the Federal District, from 2010 to 2013^22–25^.

Vigitel uses probability sampling of the adult population (≥18 years old) residing in the 26 state capitals and the Federal District. The system uses the data frame of residential telephone available annually by the main telephone companies to draw the samples. The sampling process is carried out in two steps:

- draw of 5,000 telephone lines per city, divided into subsamples of 200 lines;
- selection of a resident over 18 years of age to be interviewed.

The Vigitel weighting process consists of multiplying two factors: the inverse of the number of landline telephones and the number of adults in each household. Post-stratification weights were used so that the system results are representative for the entire adult population of each city. This weighting aims to match the estimated socio-demographic composition of the population of adults with a telephone based on the Vigitel sample in each city to the socio-demographic composition estimated for the total adult population of the same city, in the same year the survey was carried out.

The study used the question “Do you smoke?”, regardless of the number of cigarettes, frequency and duration of smoking, to estimate the prevalence of adult smokers according to the IBP in the period from 2010 to 2013.

### Geoprocessing

Using the Vigitel samples with telephone and complete address information and the interview databases with telephone number information, it was possible to include the census tract by performing a linkage with the National Register of Addresses for Statistical Purposes (*Cadastro Nacional de Endereços para Fins Estatísticos* – CNEFE) of the 2010 census^26^. At the end of processing the database, IBP information by census sector was added.

### Brazilian deprivation index

The IBP is an index of three components: the percentage of households with an income of less than half the minimum wage, the percentage of illiterate people under seven years of age, and the percentage of people with inadequate access to sanitary sewage, water and garbage disposal, without a bathroom^11^. In this way, the IPB makes it possible to highlight the inequalities of different social groups by census sector.

In the North and Northeast regions, the IBP was grouped into four categories: low, medium, high, and very high deprivation. While, in the other regions, the IBP was grouped into three categories (low, medium, and high deprivation), given the high concentration of sectors in the low deprivation category and few occurrences in the high and very high deprivation categories (supplementary material - Tables S1 and S2).

### Indirect estimation for small areas

This study used data from Vigitel and the indirect estimation method to estimate the prevalence of adult smokers by IBP in the 26 state capitals and the Federal District. This method consists of using statistical models to obtain estimates of proportions of adult smokers observed in capitals for smaller areas, such as the IBP. The logistic regression model was used to impute the smoking response variable (Y), yes (1) or no (0), in the set of census sectors without any Vigitel interview. In the construction of the model, the set of sectors with a single interview in the period from 2006 to 2013 was used. This criterion was adopted due to the similarity in the distribution of sectors without an interview in Vigitel according to the IBP (supplementary material – Table S3). The response variable (y_i_) is dichotomous, with 1 being a smoker (success) and 0 (failure) otherwise (Table S4). The covariables by census sector were taken from the 2010 census, such as the percentage of households by type of water supply, percentage of households by type of sanitary sewage, percentage of households with no male members, percentage of households with female heads of household, percentage of households with grandchildren, great-grandchildren, son-in-law or daughter-in-law, parents or stepfathers or stepmothers, percentage of households with siblings over 50 years of age, and percentage of households with one or more residents.

The general model of logistic regression^27^ is given by:


$$\log\left\{\frac{\text{π}\left(x\right)}{1-\text{π}\left(x\right)}\right\}={\text{β}}_{1}+{\text{β}}_{2}x_{2}+\ldots +\beta_{p}x_{p}$$

where:

*x* = (1,*x*_2_,…,*x*_*p*_) represents the vector of covariates;

π (*x*) is the probability that the respondent self-declares a smoker (success) given the characteristic of x;

β = (β_1_,β_2_…,β_*p*_) is the vector of model parameters.

The set of sectors with a Vigitel interview was divided into two samples in the proportion of 70% for training and 30% to validate the model to ensure that the model obtained in the first sample was robust. Logistic regression calculates the probability, between 0 and 1, that the adult in the census sector is a smoker, and, to classify the adults in the sectors as smokers or non-smokers, a cut-off point in probability is used. Thus, adults in sectors with a probability greater than or equal to the cutoff point were classified as smokers and, otherwise, as non-smokers. This cutoff point was determined by analyzing the receiver operating characteristic (ROC) curve^28^.

Multiple logistic regression models were run in Rstudio version 3.6.3 using the Tidyverse package^29^.

To assess the adjusted the model, a two-by-two classification matrix was used with four possible results: true positive (TP) denotes a response of smoking being correctly classified by the model; true negative (TN) denotes a response of non-smoking being correctly classified as non-smoking. False negative (FN) responses were classified as non-smoking, and false positive (FP) responses were classified as no smoking. The sensitivity of the model is defined by $\frac{TP}{TP+FN}$ , the specificity by $\frac{TN}{TN+FP},$ and the accuracy is measured as TP+TNTP+FN+TP+FN' .

In the joint analysis of the sectors with and without interviews, the post-stratification weight adjusted for the 2010 census population by IBP was calculated using the rake method^30^. These weights were calculated in the STATA program using the SURVWGT^31^ package, requiring sample weight information to run the package. In this study, data from population N_1_ and N_2_ extracted from the 2010 census of each region were considered to calculate the weight of the group of sectors with Vigitel interviews $\left(weight=\frac{N_{1}}{n_{1}}\right)$ and without interviews $\left(weight=\frac{N_{2}}{n_{2}}\right)$ , where N_1_ is the total number of adults in sectors with Vigitel interviews, N_2_ is the total number of adults in sectors without Vigitel interviews, n_1_ is the number of Vigitel interviews and n_2_ is the number of sectors without interviews.

The prevalence ratio of adult smokers due to IBP was calculated with the aim of comparing the groups. This ratio was estimated using the Poisson model, considering the first category as a reference. These estimates were calculated using post-stratification weights.

## RESULTS

The 65,684 census sectors in the 26 Brazilian capitals and the Federal District correspond to a population of 45,980,581 people. This corresponds to 22% of the total census sectors and 24% of the Brazilian population. Of this total of census sectors, 38,867 (58.2%) sectors had at least one Vigitel interview in the period from 2010 to 2013. Analyzing by region, the North, Northeast, and South regions had 83.1%, 81.3%, and 82.0% sectors with interviews, with a median equal to five, three, and three interviews, respectively. This shows the good spread of the Vigitel samples. While the Center-West Region presented 69.9% (median=3) and 39.2% in the Southeast. In the Southeast Region, the capitals São Paulo and Rio de Janeiro have 18,182 and 10,158 sectors respectively, both with a median equal to one interview per sector, which explains the low percentage of sectors with Vigitel interviews (supplementary material – Table S4).

In general, Vigitel's samples of residential telephones are scattered throughout the capitals, with the exception of São Paulo and Rio de Janeiro.

To illustrate the IBP, Figure 1a (Supplementary) shows the census sectors of Salvador (3,530 sectors) and Figure 1b (Supplementary) the sectors grouped by IBP: low (29.7%), medium (32.0%), high (35.0%), and very high deprivation (3.3%).

### Imputation of missing data

In the construction of the logistic regression models, the census sectors were selected with an interview in the period from 2006 to 2013 (supplementary material – Table S5). This number of sectors varied between 7 (Boa Vista) and 4,231 (São Paulo). Due to the high variability in the number of sectors per region, the number of models was reduced from 27, one for each capital, to 5 models: North, Northeast, Southeast, South, and Center-West regions (supplementary material – Table S5).

The adjusted models for the North, Northeast, Southeast, South, and Center-West regions are available in the supplementary material – Tables S6 to 10. The measures of accuracy, sensitivity, and specificity of the models obtained in the two samples showed good adequacy of the models. However, the ability of the model to classify the individual as a non-smoker, given that he is a non-smoker, was greater when compared to its specificity (Supplementary material – Table S11).

### Indirect estimation

The trend of increasing prevalence as deprivation increases was found in 16 (59.3%) of the 27 cities, indicating a positive gradient. In nine (33.3%) cities, the most deprived sectors had a higher prevalence of smokers when compared to those with less deprivation and, in the other two (7.4%), there were no differences (Tables 1 to 3).

**Table 1:** Prevalence estimate and prevalence ratio of adult smokers by city and by Brazilian Deprivation Index. Northern Region, Vigitel, 2010–2013.

Municipality	IBP	%	95%CI	PR	95%CI
Belém	Low	5.98	5.03–6.94	1.00	
	Medium	8.86	7.49–10.23	1.48	1.19–1.85
	High	14.86	12.58–17.14	2.48	1.99–3.10
	Very high	24.09	16.07–32.11	4.03	2.78–5.83
Boa Vista	Low	8.42	7.33–9.51	1.00	
	Medium	7.81	6.75–8.87	0.93	0.77–1.12
	High	12.03	9.76–14.30	1.43	1.14–1.80
	Very high	23.24	11.13–35.35	2.76	1.61–4.72
Macapá	Low	5.74	4.51–6.97	1.00	
	Medium	7.52	6.44–8.61	1.31	1.01–1.70
	High	8.83	7.69–9.97	1.54	1.20–1.98
	Very high	17.54	11.45–23.64	3.06	2.03–4.60
Manaus	Low	6.38	5.07–7.68	1.00	
	Medium	7.47	6.43–8.50	1.17	0.91–1.50
	High	9.20	7.96–10.44	1.44	1.13–1.84
	Very high	8.55	5.66–11.43	1.34	0.90–1.99
Palmas	Low	7.10	6.38–7.81	1.00	
	Medium	15.91	9.10–22.72	1.28	1.03–1.59
	High*	29.29	18.73–39.86	1.38	1.02–1.88
Porto Velho	Low	8.81	7.25–10.36	1.00	
	Medium	10.24	8.96–11.52	1.16	0.94–1.44
	High	13.73	11.34–16.13	1.56	1.22–2.00
	Very high	23.04	13.80–32.28	2.62	1.69–4.05
Rio Branco	Low	10.76	8.88–12.63	1.00	
	Medium	11.12	9.56–12.67	1.03	0.83–1.29
	High	12.86	11.15–14.57	1.20	0.96–1.49
	Very high	15.65	10.18–21.12	1.45	0.98–2.15

IBP: Brazilian Deprivation Index; CI: confidence interval; PR: prevalence ratio;

* High and Very High categories were grouped together due to the small number of interviews in the period.

**Table 2:** Prevalence estimate and prevalence ratio of adult smokers by city and by Brazilian Deprivation Index. Northeast Region, Vigitel, 2010–2013.

Municipality	IBP	%	95%CI	PR	95%CI
Aracaju	Low	6.20	5.09–7.32	1.00	
	Medium	7.16	5.88–8.44	1.15	0.90–1.49
	High	11.29	8.29–14.28	1.82	1.32–2.51
	Very high	30.58	19.94–41.21	4.93	3.33–7.29
Fortaleza	Low	7.34	6.15–8.53	1.00	
	Medium	8.92	7.56–10.28	1.22	0.97–1.52
	High	16.70	15.00–18.40	2.28	1.88–2.76
	Very high	30.20	25.57–34.82	4.11	3.29–5.14
João Pessoa	Low	7.09	6.05–8.13	1.00	
	Medium	7.57	6.29–8.85	1.07	0.85–1.33
	High	18.71	15.34–22.09	2.64	2.09–3.33
	Very high	35.93	29.80–42.06	5.07	4.04–6.34
Maceió	Low	6.91	5.67–8.16	1.00	
	Medium	6.25	4.86–7.64	0.90	0.68–1.20
	High	8.41	6.98–9.83	1.22	0.95–1.56
	Very high	11.54	8.92–14.17	1.67	1.25–2.23
Natal	Low	8.10	6.88–9.32	1.00	
	Medium	8.11	6.90–9.32	1.00	0.81–1.24
	High	10.41	8.68–12.15	1.29	1.03–1.61
	Very high	18.76	13.79–23.73	2.32	1.71–3.14
Recife	Low	12.20	10.56–13.85	1.00	
	Medium	11.04	9.23–12.85	0.90	0.73–1.12
	High	17.90	15.97–19.84	1.47	1.23–1.74
	Very high	33.17	28.85–37.49	2.72	2.25–3.28
Salvador	Low	6.83	5.74–7.93	1.00	
	Medium	6.90	5.88–7.91	1.01	0.81–1.25
	High	9.23	7.99–10.48	1.35	1.10–1.67
	Very high	22.07	15.15–28.99	3.23	2.27–4.59
São Luís	Low	6.91	5.57–8.26	1.00	
	Medium	8.09	6.22–9.96	1.17	0.86–1.58
	High	8.42	6.79–10.06	1.22	0.93–1.60
	Very high	12.35	9.21–15.48	1.79	1.30–2.46
Teresina	Low	5.03	4.04–6.03	1.00	
	Medium	7.05	5.96–8.14	1.40	1.09–1.80
	High	7.45	6.15–8.75	1.48	1.14–1.93
	Very high	10.80	7.68–13.92	2.15	1.51–3.05

IBP: Brazilian Deprivation Index; CI: confidence interval; PR: prevalence ratio.

**Table 3:** Prevalence estimate and prevalence ratio of adult smokers by region, city and Brazilian Deprivation Index. Southeast, South, and Center-West Regions, Vigitel, 2010-2013.

Region	Municipality	IBP	%	95%CI	PR	95%CI
Southeast	Vitória	Low	11.12	9.65–12.59	1.00	
		Medium	10.54	8.96–12.12	0.95	0.78–1.16
		High	12.46	7.71–17.20	1.12	0.75–1.68
	Belo Horizonte	Low	16.84	15.77–17.91	1.00	
		Medium	19.12	17.38–20.87	1.14	1.02–1.27
		High	23.56	20.36–26.75	1.40	1.20–1.62
	Rio de Janeiro	Low	21.35	20.37–22.32	1.00	
		Medium	28.20	26.63–29.76	1.32	1.23–1.42
		High	34.71	32.61–36.80	1.63	1.51–1.75
	São Paulo	Low	27.82	26.96–28.69	1.00	
		Medium	27.74	26.65–28.84	1.00	0.95–1.05
		High	37.52	35.77–39.26	1.35	1.28–1.43
South	Curitiba	Low	14.25	13.24–15.27	1.00	
		Medium	19.02	16.49–21.55	1.33	1.15–1.55
		High	31.48	23.27–39.69	2.21	1.69–2.89
	Florianópolis	Low	9.10	8.25–9.95	1.00	
		Medium	13.17	10.93–15.41	1.45	1.19–1.76
		High	21.44	10.14–32.75	2.36	1.38–4.02
	Porto Alegre	Low	16.66	15.48–17.84	1.00	
		Medium	27.71	24.88–30.53	1.66	1.47–1.88
		High	39.68	34.55–44.80	2.38	2.06–2.76
Center-West	Campo Grande	Low	9.49	8.34–10.63	1.00	
		Medium	10.69	9.43–11.96	1.13	0.95–1.33
		High	12.59	10.84–14.34	1.33	1.10–1.60
	Cuiaba	Low	11.13	9.39–12.86	1.00	
		Medium	12.06	9.70–14.42	1.08	0.84–1.39
		High	16.51	13.60–19.42	1.48	1.17–1.88
	Goiânia	Low	9.26	8.13–10.40	1.00	
		Medium	11.56	10.04–13.08	1.25	1.04–1.49
		High	17.28	14.71–19.84	1.86	1.54–2.26
	Federal District	Low	11.62	10.61–12.62	1.00	
		Medium	25.22	23.15–27.29	1.25	1.04–1.49
		High	34.80	31.51–38.10	1.86	1.54–2.26

IBP: Brazilian Deprivation Index; CI: confidence interval; PR: prevalence ratio.

In the North Region, Belém and Macapá presented a positive gradient between the prevalence of adult smokers and IBP, whose prevalence estimates were three times higher in sectors with greater deprivation when compared to those with less deprivation. Followed by Boa Vista, Porto Velho, and Palmas with 2.62 (95%CI 1.69–4.05), 2.76 (95%CI 1.61–4.72), and 1.38 (95%CI 1.02– 1.88), respectively. In Manaus and Rio Branco, no differences were detected between IBP prevalence estimates (Table 1).

In Aracaju, Fortaleza, João Pessoa, and Salvador, the prevalence of adult smokers in the most deprived sectors was three times higher than in the low ones. While in Natal, Recife, and Teresina, the prevalence ratio of adult smokers ranged between 2.15 (95%CI 1.51–3.05) and 2.72 (95%CI 2.25–4.59). In Maceió and São Luís, the prevalence ratio was 1.67 (95%CI 1.25–2.23) and 1.79 (95%CI 1.30–2.46), respectively (Table 2).

In the Southeast, South, and Center-West regions, the prevalence ratios of adult smokers ranged from 1.33 (95%CI 1.10–1.60) in Campo Grande to 2.76 (95%CI 1.38–4.02) in Florianopolis. In Curitiba, Florianópolis, and Porto Alegre, the prevalence ratios of adult smokers were twice as high in the sectors with the greatest deprivation when compared to those with the least (Table 3).

## DISCUSSION

This study used the IBP to measure intra-urban inequalities in the prevalence of adult smokers, in Brazilian capitals and the Federal District, using Vigitel data from 2010 to 2013 and the indirect method for estimation in small areas.

The study takes an ecological approach to measuring health inequalities, pointing out that the areas of greatest deprivation also had the highest prevalence of adult smokers. In Aracaju, Fortaleza, João Pessoa, and Salvador, the prevalence of smokers in very high deprivation sectors is three times higher than in low deprivation ones. The results found in the study are consistent with the literature, which points to an association between the highest prevalence of tobacco and the population with low income and education in Brazil^2,3,32^ and in other countries^33,34^.

Bernal et al.^35^ showed the external validity of the estimate of the prevalence of adult smokers calculated using the indirect estimation method on Vigitel Belo Horizonte data. This study used the Health Vulnerability Indicators (HVI) grouped into four categories to estimate the prevalence of adult smokers in each group. Similarities were found between the estimates calculated in Vigitel and in the household survey, corroborating the results found here.

The work has some limitations. First, in 14% of the Vigitel interviews, the census sectors were not identified in the linkage process. The second is related to the lack of Vigitel interviews in some sectors, mainly in those with high or very high deprivation, requiring the use of statistical models to impute missing data in these sectors. In this sense, the covariates of the model may have underestimated or overestimated the probability of the adult being classified as a smoker or not in the sector. The capitals São Paulo and Rio de Janeiro have 28 and 43% of the sectors with interviews; in these capitals, the model may have underestimated the proportion of adult smokers. Third, the use of data from the 2010 census for the construction of post-stratification weights by IBP to minimize the selection bias of Vigitel in the period from 2010 to 2013 and of the covariates of the models. Due to the long-time span of the last census, these covariates may change over time. Fourth, the joining of the Vigitel databases from 2006 to 2013 given the annual variation in prevalence (supplementary material – Table S12).

Brazil produces a lot of research data in the health area with national coverage, large regions, federation unit, metropolitan region, and capitals. However, most of these states lack health information on their population in small areas, due to the high cost of surveys of this nature. In this sense, the IBP can be used to measure intra-urban inequalities in the country.

This study contributes in the methodological aspect to the production of indicators in smaller areas and, thus, subsidize the states with this information for the formulation, monitoring, and evaluation of programs and public policies for the adequate promotion of health to combat smoking.

## References

[1] World Health Organization (2013). Global action plan for the prevention and control of noncommunicable diseases 2013-2020 [Internet].

[2] Bazotti A, Finokiet m, Conti IL, França MTA, Waquil PD (2016). Tabagismo e pobreza no Brasil: uma análise do perfil da população tabagista a partir da POF 2008-2009. Ciênc Saúde Colet.

[3] Bernal RTI, Carvalho QH, Pell JP, Leyland AH, Dundas R, Barreto ML (2020). A methodology for small area prevalence estimation based on survey data. Int J Equity Health.

[4] Costa DAS, Mingoti SA, Andrade ACS, Xavier CC, Proietti FA, Caiaffa WT (2017). Indicadores dos atributos físicos e sociais da vizinhança obtidos pelo método de Observação Social Sistemática. Cad Saúde Pública.

[5] Fleury S (2011). Desigualdades injustas: o contradireito à saúde. Psicol Soc.

[6] Abegunde DO, Mathers CD, Adam T, Ortegon M, Strong K (2007). The burden and costs of chronic diseases in low-income and middle-income countries. Lancet.

[7] Martins EF, Rezende ED, Almeida MCM, Lana FCF (2013). Mortalidade perinatal e desigualdades socioespaciais. Rev Latino-Am Enfermagem.

[8] Pampalon R, Hamel D, Gamache P, Raymond G (2009). A deprivation index for health planning in Canada. Chronic Dis Can.

[9] Januário GC, Alves CRL, Lemos SMA, Almeida MCM, Cruz RC, Friche AAL (2016). Índice de vulnerabilidade à saúde e triagem auditiva neonatal: diferenciais intraurbanos. CoDAS.

[10] Rao JNK, Molina I (2015). Small area estimation.

[11] Allik M, Leyland A, Ichiara MYT, Dundas R (2020). Creating small-area deprivation indices: a guide a guide for stages and options. J Epidemiol Community Health.

[12] Townsend P (1987). Deprivation. Journal of Social Policy.

[13] Allik M, Brown D, Dundas R, Leyland AH (2016). Developing a new small-area measure of deprivation using 2001 and 2011 census data from Scotland. Health Place.

[14] McLoone P (1994). Carstairs scores for scottish postcode sectors from the 1991 census [Internet].

[15] Chondur R, Guthridge S, Lee H (2005). Socio-economic indexes for areas (SEIFA) of administrative health districts and urban centres/localities in the Northern Territory [Internet].

[16] Fukuda Y, Nakamura K, Takano T (2007). Higher mortality in areas of lower socioeconomic position measured by a single index of deprivation in Japan. Public Health.

[17] Sánchez-Cantalejo C, Ocana-Riola R, Fernández-Ajuria A (2008). deprivation index for small areas in Spain. Soc Indic Res.

[18] Salmond CE, Crampton P (2012). Development of New Zealand's deprivation index (Nzdep) and its uptake as a national policy tool. Can J Public Health.

[19] Cabrera-Barona P, Murphy T, Kienberger S, Blaschke T (2015). A multi-criteria spatial deprivation index to support health inequality analyses. Int J Health Geogr.

[20] Vasquez A, Cabieses B, Tunstall H (2016). Where are socioeconomically deprived immigrants located in Chile? A spatial analysis of census data using an index of multiple deprivation from the last three decades (1992-2012). PLoS One.

[21] Noble M, Barnes H, Wright G, Roberts B (2010). Small area indices of multiple deprivation in south Africa. Soc Indic Res.

[22] Brasil (2011). Vigitel Brasil 2010: vigilância de fatores de risco e proteção para doenças crônicas por inquérito telefônico.

[23] Brasil (2012). Vigitel Brasil 2011: vigilância de fatores de risco e proteção para doenças crônicas por inquérito telefônico.

[24] Brasil. Ministério da Saúde (2013). Vigitel Brasil 2012: vigilância de fatores de risco e proteção para doenças crônicas por inquérito telefônico.

[25] Brasil. Ministério da Saúde (2014). Vigitel Brasil 2013: vigilância de fatores de risco e proteção para doenças crônicas por inquérito telefônico.

[26] Instituto Brasileiro de Geografia e Estatística Censo demográfico 2010 [Internet].

[27] Paula GA (2013). Modelos de regressão com apoio computacional.

[28] Habibzadeh F, Habibzadeh P, Yadollahie M (2016). On determining the most appropriate test cut-off value: the case of tests with continuous results. Biochem Med (Zagreb).

[29] R Core Team (2020). R: A language and environment for statistical computing [Internet].

[30] Cervantes IF, Brick JM, Jones ME (2002). Weighting for nontelephone household in the 2001 California health interview survey. Joint Statistical Meetings – Section on Survey Research Methods.

[31] Winter N (2002). SURVWGT: stata module to create and manipulate survey weights [Internet].

[32] Malta DC, Vieira ML, Szwarcwald CL, Caixeta R, Brito SMF, Reis AAC (2015). Tendência de fumantes na população brasileira segundo a Pesquisa Nacional de Amostra de Domicílios 2008 e a Pesquisa Nacional de Saúde 2013. Rev Bras Epidemiol.

[33] Giovino GA, Mirza SA, Samet JM, Gupta PC, Jarvis MJ, Bhala N (2012). Tobacco use in 3 billion individuals from 16 countries: an analysis of nationally representative cross-sectional household surveys. Lancet.

[34] Chen A, Machiorlatti M, Krebs NM, Muscat JE (2019). Socioeconomic differences in nicotine exposure and dependence in adult daily smokers. BMC Public Health.

[35] Bernal RTI, Malta DC, Peixoto SV, Costa MFL (2021). Validação externa da estimativa da prevalência de fumantes em pequenas áreas produzida pelo Vigitel, em Belo Horizonte, Minas Gerais, Brasil. Rev Bras Epidemiol.

